# Infantile Paralysis in a Young Adult

**Published:** 1909-03-13

**Authors:** Howard V. Mitchell


### The General Practitioner's Column.

[Contributions to this Column are invited, and if accepted will be paid for.]

### INFANTILE PARALYSIS IN A YOUNG ADULT.

By HOWARD V. MITCHELL, M.R.C.S., L.R.C.P.

Acute anterior poliomyelitis in a robust young man, aged 25 years, is sufficiently interesting to be recorded. C. R., a farm labourer, single, healthy-looking, of E——, near Lichfield, was seen two days after the onset of his illness; he complained of loss of power in the right arm and right leg. The previous history was quite good; no sore throat, influenza, syphilis, or any infectious disease. When 14 years of age the patient had an accident with an ammonia bottle, which resulted in temporary blindness, and which may explain a slight inequality of his pupils; the right was a shade smaller than the left.

After working on the farm for several days, and in wet weither, on Sunday, November 29, the patient noticed that he could not lift with his right arm. He did not, however, feel anything amiss with his right leg at the time, but says that he felt as if he had had a severe chill. Seen on the Monday by a doctor, he had complete loss of power in the right arm and right leg, and complained of a headache. As it was not possible to nurse him at his home, it was decided to remove him to the hospital at Tamworth. When received there he exhibited total motor paralysis of the right arm and right leg and partial motor paralysis of the left leg. There was no facial paralysis. Sensation was everywhere normal, and there was no bladder or rectal trouble. An aching pain was complained of around the chest and down the spine for a few days, and there was also some little difficulty in swallowing, but this soon passed off. The temperature ranged from 99° to 100°; a few days after admission it became normal and remained so. No knee or elbow jerks could be obtained, and there was no ankle clonus. The electrical reactions were tried with the galvanic current only; the muscles responded to a gentle stimulus. The pupils reacted to light and accommodation; the right was a little smaller than the left. No strabismus was present.

The paralysed limbs for the first few days were wrapped in cotton-wool and flannel and kept at rest. A small cradle was used to keep the weight of the bed-clothes off the feet. After the first week the muscles were massaged daily and the galvanic current used. After one month all the muscles of the right arm and right leg are much wasted. The patient is able to flex and extend the toes of the right leg, but is unable to extend the knee. The flexors of the arm and forearm have recovered slightly, but the extensors remain paralysed as on admission. The left leg has recovered, but is still very weak. The reflexes remain absent, and there is no change in the pupils. Some of the muscles continue to react to the galvanic current, but in others the reaction cannot be obtained. The patient complains of the joints being tender when the affected limbs are moved.

The case is interesting on account of the age of the patient, a robust young man living a healthy farm life. One can realise how much more difficult the diagnosis of acute anterior poliomyelitis must be in children, especially in those too young to speak.

